# A 40-bp Insertion/Deletion Polymorphism of Murine Double Minute2 (MDM2) Increased the Risk of Breast Cancer in Zahedan, Southeast Iran

**DOI:** 10.6091/ibj.13332.2014

**Published:** 2014-10

**Authors:** Mohammad Hashemi, Mohsen Omrani, Ebrahim Eskandari-Nasab, Seyed-Shahaboddin Hasani, Mohammad Ali Mashhadi, Mohsen Taheri

**Affiliations:** 1 *Cellular and Molecular Research Center, Zahedan University of Medical Sciences, Zahedan;*; 2 *Dept. of Clinical Biochemistry, School of Medicine, Zahedan University of Medical Sciences, Zahedan; *; 3 * Dept. of Internal Medicine, School of Medicine, Zahedan University of Medical Sciences, Zahedan;*; 4 * Genetics of Non-Communicable Disease Research Center, Zahedan University of Medical Sciences, Zahedan, Iran*

**Keywords:** Breast cancer, Murine Double Minute2 (*MDM2*), Polymorphism

## Abstract

**Background:** MDM2 (Murine Double Minute2) is an oncoprotein that inhibits the P53 activity. Overexpression of *MDM2* gene has been reported in several human tumors. In the present study, we aimed to evaluate the impact of 40-bp insertion/deletion (ins/del) polymorphism on the promoter of *MDM2* and susceptibility to breast cancer in a sample of Iranian population. **Methods:** This case-control study was carried out on 236 patients with breast cancer and 203 healthy individuals. Genomic DNA was extracted from the whole blood by the salting-out method. The 40-bp ins/del polymorphism was determined by using polymerase chain reaction. **Results: **The findings indicated that *MDM2* ins/del variant increased the risk of breast cancer in co-dominant- (odds ratio [OR] = 2.09, 95% CI = 1.14-3.85, *P* = 0.018, del/del vs. ins/ins), dominant- (OR = 1.49, 95% CI = 1.02-2.18, *P* = 0.038, ins/del + del/del vs. ins/ins), and recessive- (OR = 1.86, 95% CI = 1.03-3.34, *P* = 0.038, del/del vs. ins/ins + ins/del) tested inheritance models. The del allele increased the risk of breast cancer (OR = 1.48, 95% CI = 1.11-1.98, *P *= 0.008) compared with ins allele. **Conclusions: **Our result revealed that 40-bp ins/del polymorphism in the promoter of *MDM2* increased the risk of breast cancer in an Iranian population. Further investigations with larger sample sizes and diverse ethnicities are needed to verify our findings.

## INTRODUCTION

Breast cancer is one of the most common forms of cancers among Iranian women [1]. The exact cause of breast cancer is still unknown, but genetic factors are shown to play essential roles in the pathogenesis and progress of breast cancer [2]. The p53 transcription factor, encoded by the *p53* tumor suppressor gene, is an essential regulator of the cellular stress responses [3]. Among the genetic alterations, the tumor suppressor protein, P53, is a principal mediator of multiple cellular functions, including growth arrest, senescence, and apoptosis in response to cellular damage [4, 5]. The activity of P53 may either be inactivated or be attenuated in a vast majority of human cancers through mutations in the *P53* gene or aberrant expression of proteins acting in the P53 pathway, such as Murine Double Minute2 (MDM2) [6].

*MDM2*, coded by the *MDM2 *gene, is a key negative regulator of *P53*. Besides its directly inhibiting the transcriptional activity of P53, MDM2 also functions as an E3 ubiquitin ligase responsible for the ubiquitination and proteolytic degradation of p53 [7]. Gene expression changes induced by p53 lead either to cell cycle arrest, which enables cells to repair DNA damage, or to apoptosis [8]. Overexpression of *MDM2* is observed both in epithelial cells of transgenic mice with induced mammary carcinomas [9] and in various human tumors, including breast cancer [10, 11]. Consequently, increased levels of p53 inhibitors in tumor cells resulted in the loss of p53 function. In response to many forms of stresses, the association between p53 and MDM2 is disrupted, leading to p53 stabilization and activation [12]. 

The human * MDM2* is located on chromosome 12q14.3-15 and contains 11 exons. The *MDM2* gene has a basal promoter (P1) and an alternative promoter (P2) starting in the intron 1 [13]. The promoter P2 contains a p53-responsive element and has been shown to regulate MDM2 levels in stressed cells, while the promoter P1 functions principally in a non-stressed environment [13, 14]. Genetic variant rs2279744 (SNP309 T/G) within the intronic p53-responsive promoter of the *MDM2* has been shown to be associated with the increased affinity of the trans-criptional activator Sp1, resulting in higher levels of *MDM2* mRNA and protein. This SNP has been shown to attenuate apoptotic activity and accelerate tumor formation [15, 16]. Several studies have reported the associations between rs2279744 variant and the risk of different types of cancer [-]. 

**Fig. 1:**
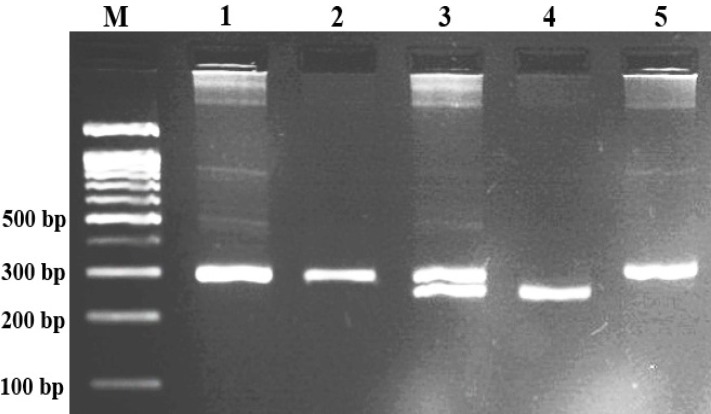
Electrophoresis pattern of PCR product of 40-bp ins/del polymorphism of MDM2 resolved by 2% agarose gel electrophoresis. M, DNA marker; Lanes 1, 2, 5, ins/ins; lane 3, ins/del; lane 4, del/del.

There is little and controversial data regarding the impact of 40-bp insertion/deletion (ins/del) poly-morphism on the constitutive promoter of *MDM2* gene and cancer risk [-]. Therefore, the present study was aimed to find out the possible association between 40-bp ins/del polymorphism in the promoter region of *MDM2* and breast cancer in a sample of Iranian population.

## MATERIALS AND METHODS

***Patients. ***This case-control study was performed on 236 histopathologically confirmed breast cancer patients and 203 age-matched women with no history of cancer of any type (as the control group) in a Southeast Iranian population. The clinicopathologic characteristics of the patients have been described in detail previously [23]. Ethical approvals for recruitment were obtained from local Ethics Committee of Zahedan University of Medical Sciences (Iran), and an informed consent was obtained from all patients and healthy individuals. Blood samples from patients and healthy controls were collected in EDTA tubes, and DNA were extracted using salting-out method as described previously [24].

***Genotyping. ***Genotyping of 40-bp ins/del polymorphism of MDM2 was performed using forward 5`-GACCACTATGTTTAAGGAAG-3` and reverse 5`-TGACTCACCTACTTTCCCAC-3` primers. PCR was performed using commercially available PCR premix (AccuPower PCR PreMix; Bioneer, Daejeon, South Korea) according to the manufacturer’s recommended protocol. The PCR cycling conditions were initial denaturation at 95°C for 5 min, followed by 30 cycles of 30 s at 95°C, 25 s at 59°C, 30 s at 72°C, with a final extension of 72°C for 10 min. The product sizes for the heterozygous ins/del production were 287 and 247 bp, respectively. The PCR products were verified onto 2% agarose gels containing 0.5 µg/ml ethidium bromide, and observed under a UV light (Fig. 1). To ensure genotyping quality, we regenotyped random samples (approximately 20% of total samples) and found no genotyping mistake.

***Statistical analysis. ***Statistical analysis was calculated using statistical package SPSS 18 software. Data were analyzed by independent sample t-test and χ2 test. The association between MDM2 ins/del variant and breast cancer was assessed by computing the odds ratio (OR) and 95% CI from logistic regression analyses. A *P* value less than 0.05 were considered statistically significant.

## RESULTS

The study group consists of 236 breast cancer patients with an average age of 47.1 ± 12.3 years and 203 healthy women with a mean age of 45.3 ± 12.8 years. No significant difference was found between the groups concerning age (*P* = 0.136).

The genotype and allele frequencies of *MDM2* ins/del polymorphism in breast cancer patients and healthy subjects are shown in Table 1. The finding indicated that ins/del variant increased the risk of breast cancer in co-dominant- (OR = 2.09, 95% CI = 1.14-3.85, *P* = 0.018, del/del vs. ins/ins), dominant- (OR = 1.49, 95% CI = 1.02-2.18, *P* = 0.038, ins/del + del/del vs. ins/ins) and recessive- (OR = 1.86, 95% CI = 1.03-3.34, *P* = 0.038, del/del vs. ins/ins + ins/del) tested inheritance models.

The deletion allele increased the risk of breast cancer (OR = 1.48, 95% CI = 1.11-1.98, *P* = 0.008) in comparison with insertion allele. The genotype frequency of the MDM2 ins/del polymorphism was tested for Hardy-Weinberg equilibrium separately in cases and controls. The genotype in controls (𝜒2 = 2.77, *P* = 0.100) but not in cases (𝜒2 = 6.88, *P* = 0.008) was in Hardy-Weinberg equilibrium. 

**Table 1:** Genotypic and allelic frequencies 40-bp ins/del polymorphism of MDM2 in breast cancer patients and control subjects

**MDM2 40-bp ins/del**	**Breast cancer** **n (%)**	**Control** **n (%)**	**OR** **(95% CI)**	***P*** ** value**
Co-dominant				
ins/ins	109 (46.2)	114 (56.1)	1.00	-
ins/del	89 (37.7)	70 (34.5)	1.33 (0.88-2.00)	0.178
del/del	38 (16.1)	19 (9.4)	2.09 (1.14-3.85)	0.018
				
Dominant				
ins/ins	109 (46.2)	114 (56.1)	1.00	-
ins/del + del/del	127 (53.8)	89 (43.8)	1.49 (1.02-2.18)	0.038
				
Recessive				
ins/ins + ins/del	198 (83.9)	184 (90.6)	1.00	-
del/del	38 (16.1)	19 (9.4)	1.86 (1.03-3.34)	0.038
				
Alleles				
ins	307 (67.2)	298 (73.4)	Ref.	-
del	165 (32.8)	108 (26.7)	1.48 (1.11-1.98)	0.008

In breast cancer patients, the ins/del polymorphism was not associated with age, tumor grade, disease stage, estrogen/progesterone receptor, and HER2/neu status (data not shown). 

## DISCUSSION

 In the present study, we investigated the impact of 40-bp ins/del polymorphism of *MDM2* on risk of breast cancer in a sample of Iranian population. The results showed that the del allele increased the risk of breast cancer in our population, and carriers of del allele were at 1.5-fold higher risk of breast cancer than those subjects with the Ins allele. Additionally, the del/del genotypes in the co-dominant and recessive models as well as the ins/del + del/del genotype in the dominant model were risk factors for developing breast cancer in our population. In contrast to our findings, Ma *et al. *[22] have found no association between *MDM2* 40-bp ins/del polymorphism and breast cancer. Hu *et al.* [21] have found that 40-bp ins/del polymorphism in the *MDM2* gene is associated with risk of lung cancer in Chinese population. Dong *et al.* [20] reported that *MDM2* ins/del polymorphism increases the risk of hepatocellular carcinoma in a Chinese population. With respect to the critical role of MDM2 in tumorigenesis, it is expectable that individuals who carry the 40-bp deletion allele may change transcription factor binding site. Higher expression of MDM2 increases the lifetime risk of developing breast cancer.

The *p53* tumor suppressor has a key role in orchestrating cellular responses to numerous types of stresses, including DNA damage and oncogene activation with apoptosis, cell-cycle arrest, DNA repair, and cell metabolism [21, 25]. Dysfunction and mutations of *p53* have been established in most human cancers, leading to a deregulated p53 activity that allows cells to proliferate and survive [26]. Many proteins regulate the activity of p53, and one of the most extensively studied regulators of p53 is MDM2 oncoprotein. The p53 activity can be regulated by MDM2 in different means, and even modest alterations of MDM2 levels can affect the p53 pathway [27]. Firstly, MDM2 directly binds to the p53 transactivation domain, consequently inhibiting its transcriptional activity. Secondly, MDM2 promotes ubiquitination and degradation of p53 by functioning as an E3 ubiquitin ligase [7, 28]. Finally, MDM2 binds p53 in the nucleus and shuttle it into the cytoplasm, promoting p53 degradation. Notably, a negative feedback loop exists between p53 and MDM2; p53 stimulates the transcription of MDM2, and in turn the MDM2 protein inhibits p53 activity [29]. 

MDM2 overexpression is detected in a number of human cancers [-]. Since MDM2 is a key component of the p53-mediated DNA-damage response, promoter polymorphism in this gene might influence this highly regulated pathway by modifying cellular MDM2 protein levels [33].

It has been proposed that functional variants in promoter regions can lead to variable gene expression levels [34]. Polymorphisms in gene promoters, involved in DNA-damage responses and apoptosis, could have an impact on individual's vulnerability to cancer development [2, 23, 35]. 

In summary, we have provided the evidence that the 40-bp ins/del polymorphism in the promoter of MDM2 gene increases the risk of breast cancer in a sample of Iranian population. However, larger sample sizes with different ethnicities are desired to validate our findings.
